# Cancer in the Testicle and Effusion in the Left Pleural Cavity

**Published:** 1887-06

**Authors:** J. A. Robinson


### CANCER OF THE TESTICLE AND EFFUSION IN THE LEFT PLEURAL CAVITY.

BY PROFESSOR J. A. ROBINSON.

[A Clinical Lecture delivered in Cook County Hospital, May 24th, 1887.]

This patient, a male, æt. 24, Frenchman, was admitted to the hospital four weeks ago on account of a scrotal tumor. At that time he was advised to have it removed, but refused, and in dread of an operation left the hospital. Day before yesterday he was again admitted on account of symptoms of disease elsewhere. You will notice the scrotum is so much enlarged as almost to hide from view the penis. You will also observe that the tumor is hard, the skin is of a purplish color and large veins course through the skin. This tumor commenced, according to the statement of the patient, eleven months ago with a swollen left testicle. It is undoubtedly of the testicle,

Observing the patient closely we see he has a cachectic appearance, is emaciated, and has great dyspnœa. On inspecting the chest we see that there is loss of motion of the left side, bulging out and effacement of the intercostal depressions, displacement of the heart to the right edge of of the sternum, and all the other signs to indicate a large effusion in the pleural cavity. The patient gives no history of pain in this side, but says he has suffered from increasing difficulty of breathing for the last three weeks. Let us inquire what will probably prove to be the character of this effusion? I believe we have good reason to anticipate that upon withdrawing some fluid we will find it to be serum, containing blood. Having now withdrawn some fluid by means of this hypodermic syringe, you see it is blood-red. This is, I think, positive proof that the effusion is due to cancer of the pleura. Cancer of the pleura is almost always secondary, and this is undoubtedly consequent upon the cancer existing in the testicle.

The patient is suffering extremely, and will be aspirated this afternoon.

In all cases where the effusion is so extensive as this, aspiration must be resorted to at once. Of course, aspiration is done in this case only for the purpose of affording temporary relief. We must pause before giving our prognosis, as it would not be considerate to discuss it in the presence of the patient, for we must remember how futile all efforts to combat cancer are. Medical science has made wonderful advancements in the treatment of disease during the last half century, but two implacable foes still face us—cancer and phthisis.

